# Persistent hiccups after acute COVID-19 successfully treated with chlorpromazine: a case report

**DOI:** 10.1186/s13256-024-04500-8

**Published:** 2024-06-19

**Authors:** Ireen Chanda Bwalya

**Affiliations:** HIV and TB Unit, Department of Infectious Diseases, Levy Mwanawasa University Teaching Hospital, Lusaka, Zambia

**Keywords:** Post-acute COVID-19, Persistent hiccup, Epidemiology, Drug treatment, Chlorpromazine, Diagnosis, Atypical, Case report

## Abstract

**Introduction:**

Hiccups are among the rare complications of COVID-19 infections. There are several published reports of persistent hiccups presenting during the acute COVID-19 period. However, there are very few published reports of persistent hiccups occurring in the post-acute COVID-19 period. Consequently, most clinicians may not be aware of this rare presentation. This case highlights an atypical presentation of persistent hiccups that manifested during the post-acute COVID -19 period that clinicians need to be aware of. The caseadds to the ever increasing body of knowledge about symptoms and signs associated with Severe Acute Respiratory Syndrome Corona Virus type 2 (SARS CoV-2) infection.

**Case presentation:**

A 27 year old male black Zambian patient presented to the emergency department of our hospital with persistent hiccup, 35 days after the initial acute episode of COVID-19. This was associated with breathlessness. There were no other symptoms. He had no history of pulmonary, gastrointestinal, neurological disease or malignancy. He did not take any alcohol or smoke. He had never used any recreational drugs. He was employed as a monitoring and evaluation officer at one of the main COVID centres in the capital. On examination, the patient was anxious. Blood pressure was 141/82, pulse rate was 95 beats per minute, respiratory rate was 26 breaths per minute, temperature was 36.8C and oxygen saturation was 97% on room air. Systemic examination was normal. Chest X-ray and abdominal ultrasonography were normal. A rapid COVID-19 antigen test, and COVID-19 Polymerase Chain Reaction (PCR) test that were done the following day were negative. All other haematological and biochemical tests, including D-dimer and C-reactive protein (CRP), were also normal. A diagnosis of post-acute COVID-19 associated hiccups was made. The patient responded well to treatment with chlorpromazine 25 mg 8 hourly. The hiccups disappeared completely after the fourth dose of chlorpromazine.

**Conclusion:**

This is one of the few published cases of COVID-19 associated persistent hiccups, occurring more than a month after the initial presentation. Most of the published cases report hiccups occurring in the acute COVID-19 period. Consequently, hiccups occurring in the post-acute COVID-19 period may not be attributable to COVID-19. This case has highlighted the need to consider post-acute COVID-19 in the differential diagnosis of persistent hiccup.

## Introduction

It has now been more than 3 years since the global pandemic of COVID-19 began. Since the pandemic began in December 2019, approximately 772, 166, 517 people worldwide have been infected [1]. COVID 19 is a viral infection caused by Severe acute respiratory syndrome corona virus-2 (SARS-CoV-2). The most common symptoms of COVID-19 are fever, cough, sore throat, headache, chest pain and shortness of breath. Other symptoms include nausea, vomiting, diarrhoea. Management of COVID-19 relies on symptomatic management, prevention of infection and respiratory support for patients with severe disease [2]. Some of the drugs that have been used in managing patients with COVID-19 include corticosteroids, remdesivir, molnupiravir and the combination of nirmatrelvir and ritonavir [3]. In addition, there are several vaccines that offer varying degrees of protection against infection with COVID-19 [4].

There are several other atypical presentations of COVID-19 that have been described in literature. Hiccups are among the atypical presentations of COVID-19. Hiccups are described as involuntary diaphragmatic and intercostal muscle contractions [5]. Persistent hiccups are those lasting more than 48 hours [6]. Some of the causes of persistent hiccups include pneumonia, central nervous system disorders such as ischemic or haemorrhagic stroke, temporal arteritis, head injury, malignancy, multiple sclerosis and syringomyelia; gastrointestinal disorders such as gastric distension, gastritis, pancreatitis, pancreatic cancer and inflammatory bowel disease; and medications such as alpha methyldopa, barbiturates, dexamethasone and carboplatin [7, 8]. Most of the cases of COVID-19 associated hiccups have been reported as occurring within 2 weeks after the initial infection [7–9]. There are very few documented reports of persistent hiccups occurring in the post-acute COVID period. This is one of the few case reports of persistent hiccups in the post-acute COVID-19 period. The post-acute COVID-19 period is the period 3 weeks after the initial acute COVID-19 episode [10].

## Case presentation

A 27 year old male black Zambian patient presented to the emergency department of our hospital with persistent hiccup of 4 days duration. The hiccup was continuous, and affected his eating and drinking. The patient also had episodes of breathlessness over the 4 days that he was having hiccup. There was no cough, fever or chest pain. There were no palpitations. There was no headache, dizziness or blurring of vision. The patient did not report any weakness of limbs or incontinence of urine or stool. There was no abdominal pain, diarrhoea or vomiting. There were no rashes or ulcerations of the skin. There was no weight loss.

He had no history of pulmonary, gastrointestinal, neurological disease or malignancy. He did not take any alcohol or smoke. He had never used any recreational drugs. He was employed as a monitoring and evaluation officer at one of the main COVID centres in the capital.

He had been diagnosed with COVID-19, 35 days prior to this presentation. During the acute episode, he had presented with cough, fever, chest pains and shortness of breath of a days’ duration. There were no other symptoms. A COVID-19 rapid antigen test was positive. Oxygen saturation was 98%. The patient’s symptoms were classified as mild. He was treated with Azithromycin 500 mg daily, Vitamin C 1000 mg daily, Paracetamol 1000 mg daily and Zinc Sulphate 20 mg daily. He self-isolated at home, and returned to work symptom free after a week.

On examination, the patient was anxious. Blood pressure was 141/82, pulse rate was 95 beats per minute, respiratory rate was 26 breaths per minute, temperature was 36.8C and oxygen saturation was 97% on room air. There was no pallor or jaundice. Neck was supple. There was no edema. There were no petechia, purpura, ecchymoses or any cutaneous lesions. Examination of oral cavity and pharynx was normal. Lymph nodes were not enlarged. Respiratory system examination was normal. There were no crepitations. Air entry bilaterally was good. Cardiovascular, gastrointestinal and neurological examinations were unremarkable.

The values of complete blood cell counts and comprehensive metabolic panel that included electrolyte, liver and kidney function was completely normal. A chest X-ray that was done was reported as being normal. An abdominal ultrasound did not reveal any sub-diaphragmatic collection of fluid or masses. The only abnormal finding was that of fatty liver. A presumptive diagnosis of COVID-19 reinfection was made, pending confirmation. There were no COVID-19 rapid tests at presentation. A sample was sent for SARS-CoV2 PCR at the reference lab. The patient was commenced on Azithromycin 500 mg daily, Zinc 20 mg daily and Vitamin C 1000 mg daily. Chlorpromazine was prescribed at a dosage of 25 mg three times a day. The patient was advised to self-isolate at home pending results of COVID-19 PCR.

A day after initial presentation, the hiccup was occurring less frequently. There was no fever, cough, chest pain or any episode of breathlessness. Vital signs were normal. Oxygen saturation was 98%. A rapid test that was done on this day was negative for SARS-CoV2. COVID-19 PCR was also negative. On the second day after commencing treatment with chlorpromazine, the hiccups stopped completely, and did not recur during the rest of the recovery period. The patient was reviewed after 2 weeks, 4 weeks and 12 weeks after resolution of hiccups. He remained completely symptom free. There was no recurrence of the hiccup.

## Discussion

Most of the cases of persistent hiccups described in COVID-19 presented early in the course of the infection [6, 7, 11]. In one systematic review, 14 out of 16 of the cases reviewed had hiccups before diagnosis of COVID-19 was confirmed [8]. To the best of my knowledge, this is one of the few published cases of persistent hiccups presenting more than a month after an initial diagnosis of COVID-19. The case by Ikitumur *et al.* [13] was similar, in that the patient developed persistent hiccups on the 13th day of hospitalization for COVID-19. In some cases, COVID-19 associated cases have subsided in the absence of specific treatment [12]. In other cases, including my present case, hiccups responded to treatment with chlorpromazine [11]. In the case that was reported by Ali *et al.* [14] the patient responded to treatment with Baclofen, a Gamma amino butyric acid (GABA) analogue which interrupts the hiccup reflex. Other treatments that have been used in the management of COVID-19 associated hiccups include metoclopramide [8]. The mean duration of hiccups is 4.6 days [8]. The pathophysiology of hiccups in COVID-19 is thought to be due to inflammation of the phrenic nerve. Most patients reported as having developed COVID-19 associated hiccups have been found to have high levels of inflammatory markers such as C-reactive protein. However, there were no signs on active inflammation, in our case as both D-dimer and C-reactive protein were normal. Karampoor *et al.* [7] report that dexamethasone can trigger hiccups in COVID-19 patients. However, our patient did not receive dexamethasone during the acute phase of treatment. The patient was not exposed to other medications known to cause hiccups, such as barbiturates, alpha methyldopa, carboplatin.

MRI of the brain was not done due to lack of resources. However, clinical neurological examination was normal. The patient did not have risk factors for ischemic or hemorrhagic stroke, such as smoking, alcohol, diabetes or hypertension. There was no history of head injury or malignancy. Abdominal ultrasound did not reveal any gastrointestinal lesions.

Lack of laboratory reagents, and lack of access to CT scan and MRI were significant limitations in this case study. Electrolytes, arterial blood gases, CT scan and MRI are not available at most health facilities. Which is why the tests were not done in this patient. Even though repeat COVID-19 test was negative, COVID 19 is the most likely cause of persistent hiccups in this previously health young man who had no history or clinical features of gastrointestinal, neurological or cardiovascular disease. False negative COVID-19 RT-PCR tests are more likely to occur in the later stages of the disease due to low viral RNA levels as the disease is cleared [9, 15].

## Conclusion

Persistent hiccups can occur in the post-acute COVID-19 period. Clinicians need to be aware of this atypical presentation, so that they can manage patients appropriately. Metoclopramide, Baclofen and chlorpromazine are effective in managing COVID-19 associated hiccups.

## Data Availability

All the relevant information related to the case is summarised in the manuscript. The original medical record is kept in the hospital registry.

## Abbreviations

ALT: Alanine aminotransferase
AST: Aspartate aminotransferase
CRP: C-reactive protein
FBC: Full blood count
GABA: Gamma aminobutyric acid
HB: Haemoglobin
PCR: Polymerase Chain Reaction
RBC: Red blood cells
SARS CoV-2: Severe Acute Respiratory Distress Syndrome Corona Virus Type 2
WBC: White blood cells
